# The impact of local supply of popular contraceptives on women’s use of family planning: findings from performance-monitoring-for-action in seven sub-Saharan African countries

**DOI:** 10.1186/s12978-023-01708-7

**Published:** 2023-11-21

**Authors:** Devon Kristiansen, Elizabeth Heger Boyle, Joseph Svec

**Affiliations:** 1 Minnesota Population Center, University of Minnesota, Minneapolis, MN USA; 2 Department of Sociology, University of Minnesota, Minneapolis, MN USA; 3 Social Sciences Department, Saint Joseph’s University, Patchogue, NY USA

**Keywords:** Unmet need for contraception, Supply and demand for contraception, Community preferences for family planning

## Abstract

Contraceptive use has substantial implications for women’s reproductive health, motivating research on the most effective approaches to minimize inequalities in access. When women prefer to limit or delay fertility but are not using contraception, this potentially reflects demand for contraception that is not being satisfied. Current literature emphasizes a nuanced integration of supply and demand factors to better understand this gap. In this research, we examine the interconnectedness of supply and demand factors both conceptually and methodologically by augmenting existing measures of local supply with a demand-side factor—community-level preferences for contraceptive methods. Using novel data from Performance Monitoring for Action (PMA) in seven sub-Saharan African countries, we test whether the available supply of locally preferred methods at nearby service delivery points (SDP) explains variation in women’s uptake of contraception beyond the more typical measure of contraceptive stockouts. Findings from logistic regression analyses (*N* = 32,282) suggest that demand and supply can be understood as tightly interconnected factors which are directly affected by local social preferences. The odds of women using modern contraception increase significantly when locally preferred methods are available, and this is true even after controlling for the availability of methods in general. The new measure tested in this research centers women and their specific desires in a manner consistent with the promotion of contraceptives as an important human right.

## Introduction

Access to contraceptives is an important human right [13, 19, 50] that is still far from fully realized. Around the world, over 200 million women who want to avoid pregnancy are not using modern contraceptive methods, and this is particularly true of poor women in the rural parts of low- and middle-income countries [18]. This is not an abstract issue given the substantial consequences for women’s maternal health and reproductive choice. Nearly a quarter of maternal mortality globally could be prevented if women wanting to avoid pregnancy but not using contraception did so [4].

Using novel data, we consider the interaction of social context and the use of contraceptives. We identify which methods are most preferred by contracepting women within local communities, evaluate the availability of those methods at nearby service delivery providers (SDPs), and consider the association of this availability with the odds women within the community who wish to limit or delay pregnancies use contraception. Our new measure, which links local supply and demand factors, constitutes an effective complement to more typical measures that focus on supply-chain disruptions in any form of contraception. We find that the presence of locally preferred methods at community SDPs increases the odds that women use contraceptives. The study provides a window into the context of supply and demand and how use is more likely to occur in conditions where contraceptive preferences match contraceptive supply.

## Background

Demographers identify “unmet need” for contraception as a woman wishing to limit or delay pregnancy and not using contraception. The problem with this conceptualization is that the survey questions used to measure unmet need fail to capture women’s actual intentions and motivations [7]. Not all women who wish to delay or limit pregnancy need contraception. For example, women may want to avoid pregnancy, but not enough to incur the social, physical, and economic burden of using contraception. Further, research has shown that potential demand for contraception, by itself, is not a particularly good predictor of future uptake of contraception [37]. Acknowledging weaknesses in the concept of unmet need, scholars have recently called for the use of the more precise term “potential demand” to replace it [45]. Following their lead, we use “potential demand” throughout this article.

The question of how to effectively make modern contraceptives available has been a long-standing debate, with some scholars emphasizing demand factors, such as the underlying motivations for using contraception, while others focus on supply factors such as the conditions of local infrastructure to make contraceptives readily available. Currently, scholars recognize that demand and supply perspectives on family planning are not mutually exclusive; both are crucial in meeting contraceptive preferences around the world [9]. However, much of the scholarship regarding contraceptive access focuses on whether women are using contraceptives with relatively little attention to the context of decision-making.

On the demand side, for a woman to choose to use a contraceptive, she must perceive that its benefits outweigh its costs in terms of resources, physical and psychological side effects, and possible social repercussions [3, 41]. Scholars often focus on child-bearing intentions as a predictor of contraceptive demand, as the desire to limit or postpone births indicates a need for effective methods to achieve those goals. Demand-side perspectives emphasize the social and economic factors that motivate fertility decisions, such as the cost of raising children, declining mortality rates, knowledge of methods, women’s decision-making, and changes in the role of children within families [6]. Such social and demographic factors frame intervention strategies that emphasize socioeconomic development as a precursor to demand for modern contraceptives. However, more recent studies distinguish between having and meeting demand. For example, a multi-country study in Sub-Saharan Africa finds that women’s desire for contraceptives are largely similar across wealth and education levels. Disparities in meeting demand emerge when wealthier and more educated women are more likely to satisfy their demand [48]. For this reason, current interventions are focusing less on “creating” demand and more on meeting existing and projected demand [16].

Community norms guide contraceptive demand, including decisions about whether and which contraceptives are socially acceptable, how and when to use them, and which potential side effects are acceptable risks. Preferences for methods are diffused through social networks that share information and normalize use [15, 17, 31]. Negative attitudes toward contraceptives within communities can significantly limit the appeal of certain methods. Across sub-Saharan Africa, perceptions of harmful side-effects and the social stigma of using contraceptives are linked with non-use [40, 44]. There is a common theme in studies of access that norms and socialized views on methods are important components of a broader ecology of decision-making [27, 32]. Demand for contraception can be understood as the response to forces that shape fertility preferences and other forces that shape desirability/acceptability of contraceptives.

There is substantial variation in family planning preferences across locations because individuals and communities differ along many dimensions. For example, injectables became very popular in Kenya in the 1990s, attributable in part to greater privacy for women whose partners may not have supported family planning [28]. In Kenya, injectables coincided with local needs—they offered effective and flexible alternatives to more long-term methods, such as IUDs and hormonal implants and could be used privately, circumventing spousal objections. On the other hand, when women living with HIV in South Africa were steered toward injectables after pregnancy, this method of family planning was associated with high rates of contraceptive discontinuation [47]. Injectables were less effective at reducing potential demand for contraception when they were provided to women in Cape Town, South Africa after pregnancy, regardless of those women’s historical method preferences. No single contraceptive method will be popular with all women or in all locations because social and historical circumstances vary widely across spaces. Rationales for using particular contraceptive methods vary due to preferences, knowledge about methods, or social obstacles [34, 40].

Based on the previous literature, our presumption is that the common usage of particular contraceptives within a community creates an environment where the most frequently used methods are well-understood and culturally acceptable and become a top choice for women turning to contraceptives for the first time or returning to contraceptives after a hiatus. Women may want to space or limit their children, but only if certain conditions are met. Contraceptives must meet those conditions to become attractive to women.

Supply-side studies of family planning represent a complementary line of research. Among other factors, such as prohibitive costs and inconvenient wait times, these emphasize the role of access to modern contraceptives in influencing fertility behaviors and preferences for usage. Increased access can crystalize latent demand and elevate family planning as a normative component of fertility decision-making processes [11, 23]. Moreover, the supply of a broad contraceptive mix, through both public and private sources, corresponds with decreased inequalities in women’s capacities to achieve their own fertility goals [2, 14]. Generalized assumptions about women’s demand in previous works can obscure whether family planning accessibility is adequately providing choice for women. In the language of reproductive rights, decision-making based on fertility preferences is paramount.

Measures of contraceptive supply are wide ranging. Data from SDPs themselves opened the door to detailed analyses of how the mix of available contraceptives and the cost of the different methods were associated with greater uptake of contraceptives (e.g., [43, 49]). These data also make it possible to study stockouts, situations in which an SDPs report typically carrying a method but being out of it on the day of the interview and/or during a period immediately prior to the interview (e.g., [33, 51].

Our analysis offers further information to policymakers by incorporating more detail, focusing on the sustained availability of contraceptives that are most popular among women within communities. Contraceptive supply, even a highly diversified supply, may still fail to meet local demand if there are shortages in specific desired methods. Stockouts are a good proxy for supply but can lead to inaccurate estimations of supply failures if method preferences are not accounted for. Specifically, stockouts do not capture all shortages in family planning methods because facilities that never carry specific methods or carry mostly unpopular methods are not considered in the measure. For example, pharmacies are unlikely to ever stock intra-uterine devices (IUDs), so those SDPs will not report stock-outs of IUDs. However, a region with zero stockouts might still have a shortage of IUDs. Previous studies that focus on counting stockouts in any contraceptive method may miss effects that occur when there is limited access to locally preferred family planning methods.

To address this, we move beyond a traditional supply/demand dichotomy to consider how links between supply and demand shape women’s abilities to plan their families. Our operationalization considers the extent to which locally preferred methods match local SDP supply. This measure captures the extent to which individuals can realize their family planning preferences within local service environments. Theoretically, supply and demand of popular modern methods should be in sync, but this assumes perfect market conditions, which rarely exist in the real world. Empirically—as we show below—supply and demand can become decoupled, and this has important implications for women’s inability to attain their family planning goals.

## Data and methods

The connection between SDPs’ provision of locally preferred contraceptive methods and women’s ability to satisfy their family planning desires is the central contribution of this research. PMA data allowed us to directly connect information from individual women, their communities, and their local service providers.

### Data

We used IPUMS Performance Monitoring for Action (PMA) [1, 8, 12, 36] to study factors influencing women’s ability to satisfy their contraceptive needs. The PMA surveys drew nationally representative samples using a multistage, clustered sampling design. Enumeration Areas (EA) contained approximately 200 households each, and 33 to 44 households were randomly selected for a household roster interview. Women between the ages of 15 and 49 were then identified from the household roster survey and asked detailed questions about their family planning use and fertility, among other topics. Service Delivery Points (SDP) are facilities that potentially provide family planning methods, such as hospitals, clinics, and pharmacies. SDPs were selected into the PMA sample if their catchment area included a sampled EA. One respondent for each SDP was selected to answer a set of questions regarding facility characteristics and family planning service provision.

### Sample

For our analysis, we drew on PMA samples from seven sub-Saharan African countries: Burkina Faso [21], Cote d’Ivoire [20], Ethiopia, Ghana [26], Kenya [22], Nigeria, and Uganda [29]. All countries had representative PMA surveys from both 2016 and 2017 except Cote d'Ivoire, which was surveyed in 2017 and 2018. The sample included 32,282 women of childbearing age (15–49) who were at risk of pregnancy and did not wish to have a child in the next two years. Women were considered at risk of pregnancy if they were sexually active, and were not currently pregnant, infecund, or menopausal. Women who met the selection criteria comprised 41% of the total PMA sample of women of childbearing age (see Table 1). The percentage of women included in the analytic sample varied somewhat by country, ranging from 34.8% in Nigeria to 53.7% in Kenya.

**Table 1:** Analytic sample selection by country

	Percent of sample in analytical sample (total)	Women in analytical sample	Total sample of surveyed women
Burkina Faso	44.3%	3013	6808
Ethiopia	36.4%	5463	15,002
Ghana	43.2%	3473	8040
Kenya	53.7%	6382	11,886
Nigeria	34.8%	7868	22,615
Uganda	46.3%	3693	7977
Cote d'ivoire	42.8%	2390	5582
Total		32,282	77,910

### Contraceptive use

A complementary concept to unmet need is “demand satisfied.” A woman is considered to have her need for contraception satisfied if she did not want to have a child within the next two years, was at risk of pregnancy, and was using a contraceptive at the time of the survey. It is important to note that demand satisfied raises the same conceptual problems as unmet need—survey questions ask for women’s own ideas about whether they wanted contraceptives or whether that demand was satisfied. For example, a woman may be considered having her demand satisfied even if she is using a contraceptive method she finds unsatisfactory [45]. While acknowledging this weakness, we use “demand satisfied” throughout this article to avoid the more precise but cumbersome term “women who say they do not want to get pregnant and are using contraception.”

We used a dichotomous measure of demand satisfied; demand satisfied was coded 1 and demand not satisfied was coded 0. We specifically focus on the potential demand for modern contraceptives, allowing us to maximize comparability across multiple countries in Sub-Saharan Africa.

### Measuring contraceptive supply

The women in our sample were matched with aggregated SDP survey data calculated for their enumeration areas. The PMA teams identified between one and nine SDPs in each EA, with most EAs having 1–3 public and 1–3 private SDPs. Among SDPs in the sample, the majority were defined as either health centers (41%), hospitals (21%), or pharmacies/drug shops (19%).

*Identifying the most popular methods* In the PMA surveys, women were asked whether they or their partners did something, or used any method, to delay or avoid pregnancy currently or within the past 12 months. If they responded affirmatively, they were asked the method used and if that was their preferred method of contraception. Aggregating the responses of women who reported using their preferred modern contraceptive method, we determined the methods used most commonly in each EA. In calculating of the most popular methods, we distinguished between the most used and the most preferred methods by only including the responses of contracepting women who reported they were using their preferred method. In addition to being more precise, this approach also limited potential endogeneity in the multivariable analysis, that is, correlation between an independent variable and the error term.^1^

The surveys allowed multiple methods to be reported; we tallied the most effective method used by each woman to construct our aggregate measures. We focused our analysis on contraceptive methods that could be provided through health personnel or commercial outlets, and did not include behavioral methods such as rhythm, lactation, and withdrawal. The latter may be made more effective with training or instruction, but do not face risks of stock-outs within SDPs. On average, just under half of the women in each EA were using the same popular method. Generally, a large majority (average across EAs = 70.0%) were using one of the top two methods. For this reason, for the most preferred methods, we focused on the top two.

*Measuring supply of the most common methods* In the surveys of SDPs, interviewers asked which contraceptive methods the facility usually provided. For each method reported, the interviewer noted if the method was in stock at the time of the interview. We created a dummy variable for each facility in the SDP data indicating whether it was currently in stock of at least one of the two most-commonly used methods in its EA, then aggregated the dummy variable at the EA level to arrive at a count of facilities that were in stock of at least one of the EA’s two most commonly used methods.^2^ We attached the aggregate variable to the female records.

*Number of facilities* We included the number of facilities in each EA as a control for general modern-contraceptive supply. The count of facilities ranged from 0 to 9 facilities and included all types of facilities. In these data, most women (about 75%) resided in EAs with 1 to 3 facilities.

*Traditional stockout measure* To compare our new measure of popular-contraceptive supply to previous measures, we also estimated the effect of the number of facilities in each EA with a stockout of *any* family planning method on demand satisfied—a more traditional stockout measure. A stockout occurred when any method that was usually provided by the facility was not in stock on the day of the interview. More SDPs in an EA provide more risk that at least one facility will have a stockout; hence the importance of including total number facilities in our models as a control.

### Demographic control variables

To isolate the effect of contraceptive supply on demand satisfied, we controlled for several demographic characteristics: the number of children ever born, the woman's age, her age squared (to capture a potential curvilinear relationship), and her marital status and education level. We also considered household characteristics, including urban/rural status and wealth quintile. Finally, we included both the country and the year of the survey as controls.

### Statistical approach

To test associations, we utilized a series of pooled country logistic regressions which include fixed effects for country and year of the survey, in addition to robust standard errors clustered at the primary sampling unit (EA). All logistic regression models predicted the outcome of having demand for contraception satisfied on all covariates listed above. We used the denormalized weight constructed by IPUMS PMA called POPWT, which multiplied each observation's survey weight by the ratio of the target population (the number of women aged 15 to 49) in each country for that year, divided by the sum of all individual weights from the sample.

As an additional assessment of contraceptive access conditions, and to reduce potential endogeneity, we included lagged variables for each of the facilities measurements (number of facilities, stock-outs, and popular methods available) to check the robustness of the findings. These lagged variable models resulted in a reduced analytic sample (*N* = 11,109) while assessing the association of satisfied demand on stock-outs and popular methods from previous waves. Estimating current levels of satisfied demand for contraceptives on previous levels of stock-outs and popular methods access mitigated some of the potential for coefficient bias and was compared with current measure analyses.

## Results

**Fig. 1:**
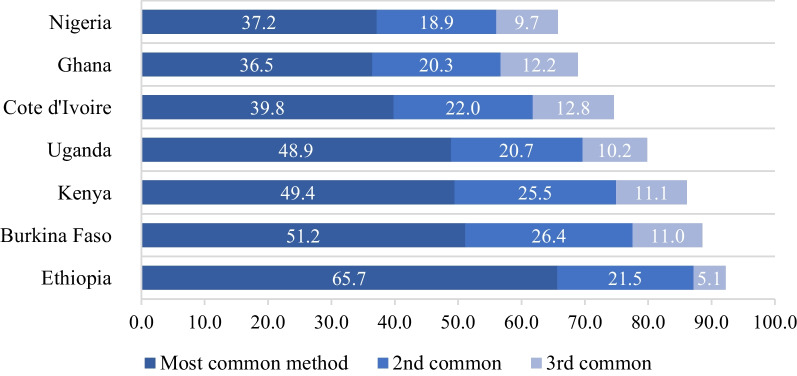
Percent of women using family planning using top 3 common methods in EA

There was wide variation in the most popular methods across communities. Figure 1 displays, for each of the seven countries in our analysis, the percentage of EAs with each possible combination of popular methods. The bottom axis identifies the most common method; the left axis lists the second most common method; each cell represents one combination of the first and second most common methods. Darker cells indicate that a relatively high percentage of EAs within the country fall into that category.

In the data, the most popular modern contraceptives overall were injectables, implants, the pill, and male condoms. In Ethiopia, the most popular method was injectables [35]. Similar use patterns for injectables were observed in Kenya and Uganda. Implants were especially popular within Burkina Faso. No methods dominated in either Cote D’Ivoire or the surveyed EAs in Nigeria, suggesting there was more variability in women’s contraceptive preferences within these countries. That said, methods of choice in Cote d’Ivoire tended to include pills and condoms, and to a lesser extent, injectables and implants. Short-acting methods, such as pills and condoms, are available at private sector service delivery points and therefore do not need to be distributed by highly trained health care providers.

**Fig. 2:**
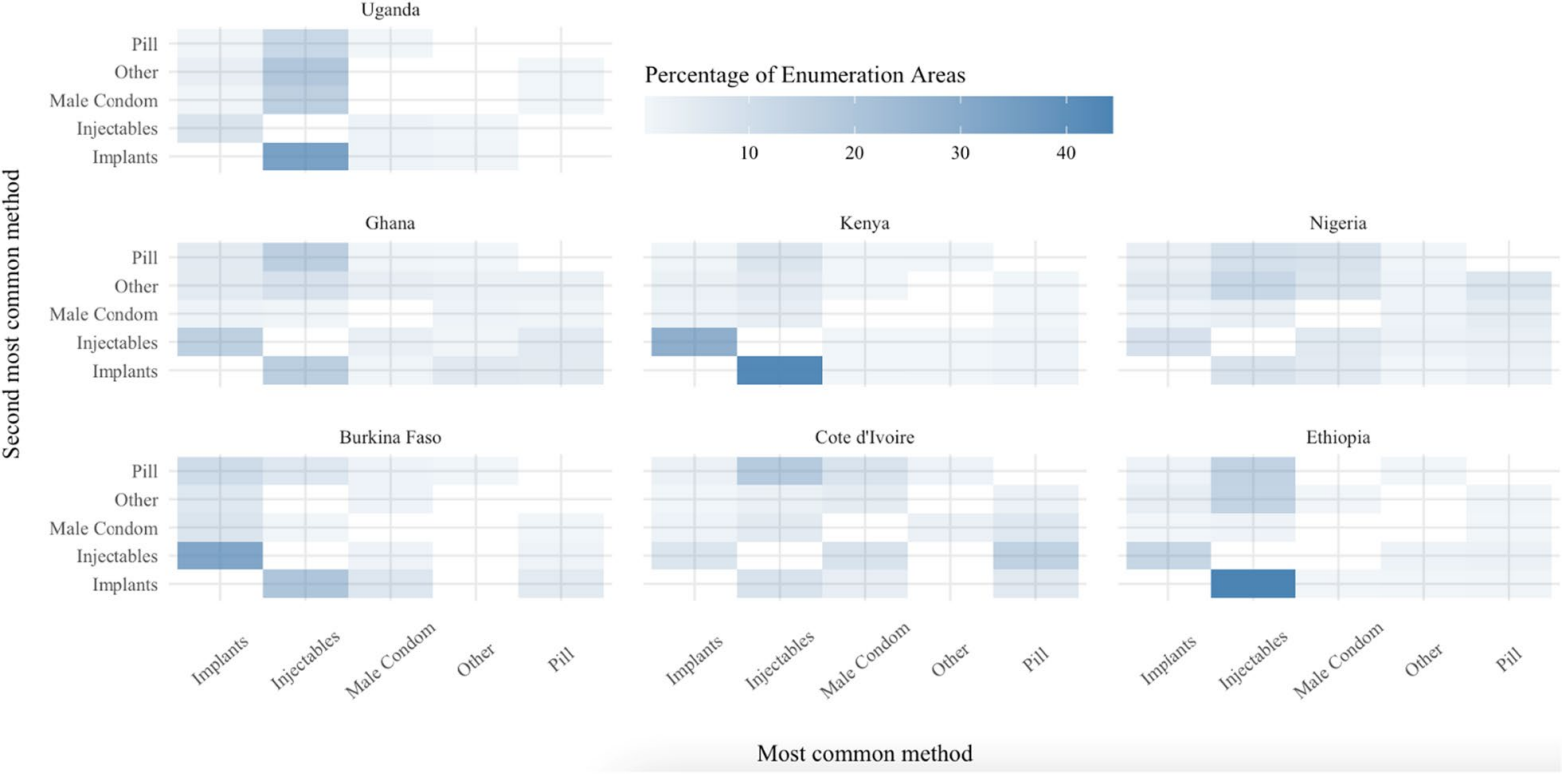
Most common contraceptive combinations at the enumeration area level

Except in Cote D’Ivoire, the pill was rarely the most common method in an EA, but it was the second-most common in several countries. The popularity of male condoms was likewise rather variable. Across the pooled sample, injectables were the most preferred method but, in nearly half of EAs, implants, pills, or male condoms were more popular. Figure 2 shows that on average the percentage of women using one of the top two methods varied from 56.1% in Nigeria to 87.20% in Ethiopia. Injectables were preferred by more women in most of the EAs studied but, in nearly half of the EAs, implants, pills, or male condoms were more popular than injectables. Overall, the notable variations in Fig. 1 indicate the importance of considering types of popular methods when measuring contraceptive supply.

Table 1 shows the percentage of women with a demand for contraception that had been satisfied. Within our sample, the percentage of women with demand satisfied ranged substantially across countries from approximately 49% in Nigeria to 80% in Kenya. Across both survey rounds, the percentage of demand satisfied remained largely stable. A notable exception was Burkina Faso. In that country, demand satisfied increased from 51% of women in 2016 to 64% of women in 2017. Burkina Faso saw significant increases in women’s use of contraceptives for spacing, from 20% in 2016 to 25% in 2017, representing the only instance in which substantial differences between rounds were observed.

In Table 2, we show descriptive statistics for women in the pooled sample. The average number of children was 3.24 per woman, and the average age was 29.6 years old (with a range of 15 to 49). In this sample, most women were married (81.0%), whereas 14.0% were never married women and 5.0% were widowed, divorced, or separated. Overall, a plurality of women had at least a primary education (36.9%) while nearly a quarter of women either had never attended school (24.8%) or had a secondary education (27.8%). Approximately 45% of women in this sample lived in urban areas.

**Table 2:** Descriptive statistics (*N* = 32,282)

	Mean/%	SD	Min	Max
*Service delivery providers*				
Number of facilities	2.38	1.47	0	9
Stock-outs of any method	0.95	1.07	0	7
Number of facilities with popular methods	1.86	1.30	0	8
*Women wishing to space or limit*				
Number of children ever born	3.22	2.55	0	19
Age (in single years)	29.8	8.03	15	49
Age-squared	9.53	5.03	2.25	24.01
Marital status				
Never married (omitted)	14.7%		0	1
Married/cohabiting	80.1%		0	1
Widowed/divorced/separated	5.2%		0	1
Education level				
Never attended (omitted)	24.8%		0	1
Primary/middle school	36.9%		0	1
Secondary/post-primary	27.8%		0	1
Tertiary/post-secondary	10.5%		0	1
Wealth quintiles				
Lowest	19.3%			
Lower	19.0%			
Middle	18.1%			
Higher	18.9%			
Highest	24.8%			
Urban resident	45.3%		0	1

Turning to the contraceptive supply environment, the average number of SDPs in each enumeration area was 2.45. Out of 2080 EA-year combinations, there were only 130 with no SDPs, or less than 7% of the sample. Rural EAs were slightly less likely to have zero SDPs compared to urban areas. Facilities tended to stock the most popular methods of contraceptives. On average, 1.82 facilities (out of the average 2.45) in each EA were providing at least one of the two most popular methods. While this suggests, as expected, that service delivery points are generally responsive to demand, it also reveals that mismatches between supply and demand are not uncommon.

The logistic regression results predicting the fulfillment of women’s demand for contraceptives are provided in the Table 3 below. Model 1 includes a very basic measure of modern contraceptive supply—the number of facilities that provide contraceptives within each woman’s enumeration area. Model 2 adds an additional piece of information with the number of stock-outs of any method. Model 3 considers our new measure of stock-ins of popular methods, and Model 4 combines all the different measures of supply.

**Table 3:** Logistic regression: Log odds of demand satisfied by popular methods in stock, denormalized weight

	Model 1	Model 2	Model 3	Model 4	Model 5	Model 6
Number of facilities	1.06**	1.09**	0.99	1.01		
	(1.02–1.11)	(1.03–1.14)	(0.93–1.05)	(0.95–1.07)		
Stock-outs of any method		0.94*		0.93*		
		(0.88–1.00)		(0.87–0.99)		
Number of facilities with popular methods			1.12**	1.12***		
			(1.04–1.19)	(1.05–1.20)		
Lag: Number of facilities					1.05	0.98
					(0.97–1.13)	(0.90–1.07)
Lag: Stock-outs of any method					1.03	
					(0.92–1.15)	
Lag: Number of facilities with popular methods						1.12*
						(1.01–1.24)
Number of children ever born	0.88***	0.88***	0.88***	0.88***	0.92***	0.92***
	(0.86–0.90)	(0.86–0.90)	(0.86–0.90)	(0.86–0.90)	(0.89–0.96)	(0.89–0.96)
Age (in single years)	1.25***	1.25***	1.25***	1.25***	1.25***	1.25***
	(1.21–1.29)	(1.21–1.29)	(1.21–1.29)	(1.21–1.29)	(1.19–1.32)	(1.19–1.32)
Age-squared	0.75***	0.75***	0.75***	0.75***	0.76***	0.76***
	(0.72–0.79)	(0.71–0.79)	(0.71–0.79)	(0.71–0.79)	(0.70–0.83)	(0.70–0.82)
Marital status						
Never married (omitted)						
Married/cohabiting	0.74***	0.75***	0.75***	0.75***	0.58***	0.58***
	(0.63–0.88)	(0.63–0.88)	(0.63–0.89)	(0.64–0.89)	(0.45–0.75)	(0.46–0.75)
Widowed/divorced/separated	1.08	1.09	1.09	1.10	0.73	0.74
	(0.85–1.38)	(0.86–1.38)	(0.86–1.39)	(0.87–1.40)	(0.50–1.07)	(0.50–1.09)
Education level						
Never attended (omitted)						
Primary/middle school	1.74***	1.74***	1.73***	1.73***	1.86***	1.84***
	(1.55–1.95)	(1.55–1.95)	(1.54–1.94)	(1.54–1.94)	(1.53–2.26)	(1.52–2.24)
Secondary/post-primary	2.09***	2.09***	2.09***	2.09***	2.13***	2.12***
	(1.83–2.39)	(1.83–2.39)	(1.83–2.38)	(1.83–2.39)	(1.71–2.66)	(1.70–2.64)
Tertiary/post-secondary	2.67***	2.67***	2.63***	2.64***	2.85***	2.81***
	(2.22–3.20)	(2.22–3.21)	(2.20–3.16)	(2.20–3.16)	(2.11–3.85)	(2.08–3.79)
Wealth quintiles						
Poorest (omitted)						
Poor	1.32***	1.33***	1.33***	1.34***	1.26*	1.26*
	(1.15–1.52)	(1.16–1.52)	(1.16–1.52)	(1.17–1.53)	(1.01–1.56)	(1.01–1.56)
Middle	1.48***	1.49***	1.48***	1.49***	1.36*	1.36*
	(1.27–1.72)	(1.28–1.73)	(1.27–1.72)	(1.29–1.73)	(1.08–1.72)	(1.07–1.72)
Rich	1.81***	1.82***	1.82***	1.83***	1.69***	1.71***
	(1.53–2.12)	(1.55–2.14)	(1.55–2.14)	(1.56–2.15)	(1.32–2.16)	(1.33–2.19)
Richest	2.15***	2.16***	2.18***	2.20***	1.87***	1.89***
	(1.80–2.56)	(1.82–2.58)	(1.83–2.59)	(1.84–2.61)	(1.39–2.51)	(1.40–2.55)
Urban resident	1.05	1.03	1.03	1.02	1.14	1.11
	(0.91–1.21)	(0.90–1.19)	(0.90–1.18)	(0.88–1.17)	(0.93–1.40)	(0.91–1.37)
Constant	0.03***	0.03***	0.02***	0.02***	0.05***	0.05***
	(0.01–0.05)	(0.01–0.05)	(0.01–0.04)	(0.01–0.04)	(0.02–0.11)	(0.02–0.11)
Enumeration areas	1457	1457	1457	1457	704	704
Observations	32,282	32,282	32,282	32,282	11,109	11,109

^***^p < 0.001, **p < 0.01, *p < 0.05, 95% Confidence Intervals in Parentheses

All models include the socioeconomic and demographic characteristics of individual women as covariates. Focusing on Model 1, the results indicate that each additional child born corresponded with 12% lower odds (OR = 0.88) of demand for contraceptives satisfied. We also observe that the association of age with demand satisfied was curvilinear, as indicated by the positive association of age (OR = 1.25) and the negative association of age squared (readjusted to units of 100; OR = 0.75) with the dependent variable. In combination, these associations suggest that, among women who wanted to avoid pregnancy, the youngest and oldest women were less likely to be using contraceptives than those in the midrange of the age distribution. This is consistent with previous findings that younger women may be unfamiliar with contraceptives or may view them as unnecessary if they are not in a stable sexual relationship [10, 46].

We also observe that married women, relative to never married women, had 25% lower odds of demand satisfied for contraceptives (OR = 0.74). In terms of socioeconomic statuses, higher levels of education and wealth were positively related with demand satisfied. Compared to the lowest levels of education, women with primary education have 74% increased odds (OR = 1.74), women with secondary education had 109% increased odds (OR = 2.09) and women with tertiary or post-secondary education had 167% increased odds (OR = 2.67) of having demand satisfied. Furthermore, all wealth quintile categories corresponded with successively higher odds of demand satisfied, relative to the poorest wealth quintile. Lastly, we do not observe any difference in demand satisfied between urban and rural residents, all else controlled. Across models, the association of economic and demographic measures with demand satisfied are consistent.

In Model 1 the number of facilities, ranging from 0 to 9, corresponds with higher odds of having demand satisfied; each additional facility corresponded with 6% increased odds (OR = 1.06). This means, for example, that women who lived in contexts where there were three facilities had 21% higher odds of demand satisfied than women who lived in contexts with no SDPs. Model 2 reports the results for stockouts in any method, controlling for the total number of SDPs. The number of stockouts is linked with reduced odds of women reporting demand satisfied for contraceptives (OR = 0.94). The findings are consistent with earlier studies showing that disrupted supply chains are associated with more women being unwilling or unable to act on their fertility preferences using modern contraceptives.

In Model 3, we introduce the presence of facilities with popular family planning methods in stock, controlling for the total number of SDPs in an EA. We observe that each additional facility with at least one of the two most popular methods corresponded with 12% increased odds (OR = 1.12) of women having demand satisfied. In this model, the total number of facilities in an EA was not associated with demand satisfied.

Model 4 demonstrates the relative independence of the stockout and stock-in measures. When both are included simultaneously in the logit models, along with a count of facilities, their independent effects are largely unchanged. Each additional stockout in an EA was associated with 7% lower odds of a woman in that EA having demand satisfied (OR = 0.93); while each additional stock-in of one of the two most popular methods was associated with 12% higher odds of this outcome (OR = 1.12). Taken together, the results support our expectation that stocking the most popular methods, and not simply the overall method mix, is an important factor for SDPs and policymakers to consider.^3^

Model 5 and Model 6 examine the association of satisfied demand for contraceptives on the lagged measures of facilities contexts. These lagged models, which include the previous wave number of facilities, stock-outs of any method, and number of facilities with popular methods, culminate in a reduced sample (*N* = 11,109) and include all current controls. We observe in Model 5 that while all control measures’ associations are similar to the findings of previous Models 1 through 4, there is no significant association between satisfied demand for contraception and previous wave levels of numbers of facilities and stock-outs. However, in Model 6, we find that the previous wave levels of the number of facilities with popular methods corresponded with 12% increased odds of satisfied demand. The contrast between past levels of stock-outs and availability of popular methods highlights the relative robustness of popular methods as a predictor of current levels of contraceptive demand satisfaction.

These results highlight how a particular emphasis on stock-outs may underestimate the extent of demand satisfied attributed to supply contexts. As we do not observe any partial mediation in Model 4, the results suggest the importance of both avoiding stockouts (providing a range of methods) and ensuring stock-ins of the most-commonly used methods. The lagged models further highlight how current contraceptive demand outcomes may also be more sensitive to historic availability of common methods rather than supply shocks more generally. The independence of coefficients at current levels on the one hand suggests that women’s ability to meet her contraceptive preferences are enhanced when local contexts experience fewer disruptions to stock more generally while the availability of preferred methods may have more long-term immediate and longer-term effects.

## Discussion

Women’s empowerment frameworks postulate that women’s goal attainment and decision-making processes are informed by the resources and capabilities at their disposal (see Kabeer [25]). Agency, defined as “the ability to define one’s goals and act upon them*”* (Kabeer [25]: 438), is the mechanism through which options are translated into action. In the family planning context, policymakers have taken an important step toward enabling women to exercise agency by ensuring that contraceptives are available to women who desire family planning. Yet, local community preferences for particular contraceptive methods are not systematically incorporated into models designed to assess women’s ability to reach goals of delaying or foregoing pregnancies. In this study, we considered the extent to which the supply of locally popular contraceptive methods provided by facilities are associated with increases in contraceptive use among women wishing to limit or delay fertility. In analyzing PMA data, we find that women who live in areas where more facilities supply one of the top two most-preferred contraceptive methods have higher odds of having their demand for contraception satisfied. This suggests that family planning strategies should more explicitly integrate community preferences into service provision.

The data show that there is tremendous heterogeneity of popular methods across countries, which highlights the importance integrating local preferences into analyses of contraceptive use among women who wish to delay or limit pregnancies. For example, the popularity of injectables in Ethiopia can in part be explained by a government-initiated, community-based contraception-distribution program in which health extension workers reached out to underserved rural communities and were often trained to administer injections. With a recent history of violence and instability, Cote d’Ivoire has an insufficient number of trained health care personnel and contraceptive service provision is limited [30], which may explain why pills and condoms—methods that do not require professional aid—tended to be most popular in communities there. More generally, the popularity of implants has increased rapidly in recent years in Sub-Saharan Africa. Implants are seen as convenient, effective, and easily reversible [24]. Moreover, implants can be used clandestinely which can help maintain anonymity in family planning practices for individuals. The various political, economic, and historic factors that contextualize methods of choice echo throughout the pattern of use and preferences. Thus, awareness of such factors in models of supply may be critical to meeting the family planning demands of women.

Across national contexts in sub-Saharan Africa, our work highlights the promise of a more nuanced measure of contraceptive supply that considers both the popularity of methods within local communities and the availability of those methods in local service delivery providers. Net of other contextual factors, such as the number of facilities and individual economic characteristics, stock-ins of locally preferred methods are consistently associated with increased odds of demand satisfied for contraception. This is evidence of the need to move beyond a one-size-fits-all approach to the study and delivery of contraceptive supply. A steady supply of the “wrong” methods may not provide women with the resources needed to enact their contraceptive preferences. This is not to say that facilities should only supply popular methods, which may still be inadequate to address all women’s needs; our findings suggest that a broad contraceptive mix is also important.

Thus, we do not mean to suggest that other measures of contraceptive supply are unimportant. Our analyses show that general stockouts are negatively associated with demand satisfied, counterbalanced by positive associations with the pure number of facilities. Beyond service contexts, increased supplies of modern contraceptives do not always translate into access, as numerous other family and social factors can impinge on contraceptive use and access. Research across Sub-Saharan Africa find that a lack of interpersonal communication or joint decision-making between partners about fertility preferences can hinder access [5, 38, 39, 42] as well as financial and location barriers [17]. Similarly, we find that demand satisfied is associated with education, where women with higher levels of education have higher odds of having demand satisfied. Likewise, women with low socioeconomic statuses have lower odds of having demand satisfied. While these findings do not obviate the importance of facility characteristics, they do highlight the persistent wealth inequalities in contraceptive access.

This research is not without limitations. Women’s preferences for particular contraceptives may be constrained by what is currently available. Women reporting that they are using their preferred method may not be fully informed about all possibilities. Fortunately, the PMA data show a wide variety of contraceptive method choices within most EAs, suggesting that many women who are contracepting have many options. We would ideally incorporate more on the quality of care provided by SDPs. There is PMA data on this subject, but the relevant questions are only asked of women who are currently using a contraceptive method. That means it is impossible to identify women who are not using contraception because they had a bad experience with, or were turned away by, a provider. Further, while family planning decisions are not always made individually, partner dynamics in family planning decision-making are outside of the scope of this research.

An additional limitation of this research is that the data do not directly connect individuals with specific facilities. Some women may travel outside their enumeration areas to access their preferred contraceptive method, but we cannot control for that in our models. Traveling to access preferred methods could therefore be biasing our coefficient estimates. Finally, access to contraceptives at nearby facilities is just one aspect of supply factors that can impede women’s ability to use contraception. Our analysis does not consider other supply-side factors, such as the expense of contraceptives or difficulty gaining access to service delivery providers. We hope that future data and analyses will address these issues.

Our results point to the importance of locally sensitive measures of supply. Attention to local preferences can catalyze demand for existing and popular contraceptive methods, limit oversupplies of methods that are infrequently used, and promote local buy-in for the service environment. The new measure tested in this research centers women and their specific desires in a manner consistent with the promotion of contraceptives as an important human right.

## Data Availability

Data are accessible at IPUMS PMA.

## Footnotes

1 The sample included 32,282 women of childbearing age (15–49) who were at risk of pregnancy and did not wish to have a child in the next two years.

2 We considered assessing whether the top three methods were available instead of the top two. We discovered that approach was problematic because the third most popular method was often much less popular than the other two; sometimes there was no third-most-popular-method at all.

3 In a robustness check, we ran separate models for each country. The direction of the stock-in and stock-out effects were consistent across all countries, although the associations were not statistically significant in most models (due to the smaller sample sizes).
